# Effect of Sleeve Gastrectomy on Proprotein Convertase Subtilisin/Kexin Type 9 (Pcsk9) Content and Lipid Metabolism in the Blood Plasma and Liver of Obese Wistar Rats

**DOI:** 10.3390/nu11092174

**Published:** 2019-09-10

**Authors:** Ewa Grzegorczyk, Monika Książek, Krzysztof Kurek, Bartłomiej Łukaszuk, Mariusz Rosołowski, Agnieszka Paszko, Michalina Krzyżak, Małgorzata Żendzian-Piotrowska

**Affiliations:** 1 Department of Hygiene, Epidemiology and Ergonomics, Medical University of Bialystok, 15-222 Bialystok, Poland; ewa.grzegorczyk@umb.edu.pl (E.G.); monika_ksiazek11.89@o2.pl (M.K.); agnieszka.paszko@o2.pl (A.P.); michalina.krzyzak@umb.edu.pl (M.K.);; 2 Department of Gastroenterology and Internal Medicine, Medical University of Bialystok, 15-276 Bialystok, Poland; mariusz.rosolowski@umb.edu.pl; 3 Department of Physiology, Medical University of Bialystok, 15-222 Bialystok, Poland; bartlomiej.lukaszuk@umb.edu.pl

**Keywords:** sleeve gastrectomy, obesity, proprotein convertase subtilisin/kexin type 9, free fatty acids, diacylglycerides, triacylglycerides

## Abstract

Nowadays, obesity and its complications are heavy burdens to western civilization. Surgical procedures remain one of the available therapies for obesity and obesity-associated diseases treatment. Among them, sleeve gastrectomy is the most common bariatric procedure. Despite the well-established fact that sleeve gastrectomy results in significant weight loss, some of its other divergent effects still need to be established. To fulfill this knowledge gap, we examined whether sleeve gastrectomy affects lipid metabolism in the plasma and liver of obese rats. We demonstrated that chronic high-fat diet feeding led to an increment in the level of Proprotein Convertase Subtilisin/Kexin (PCSK)—a regulator of plasma cholesterol concentration—in the liver, which was decreased after the gastrectomy. Moreover, we noticed significant increases in both plasma and liver contents of free fatty acids, diacylgycerides and triacylglycerides in the obese animals, with their reduction after the bariatric surgery. In conclusion, we revealed, presumably for the first time, that sleeve gastrectomy affects lipid metabolism in the liver of obese rats.

## 1. Introduction

Nowadays obesity and its complications are a considerable public health problem around the world. A large percentage of individuals with morbid obesity suffer from insulin resistance and dyslipidemia. Moreover, they are characterized by an increased risk of developing obesity-related diseases, including diabetes, cardiovascular disease (CVD) and several types of cancer [1]. The ‘weight’ of the discussed problem stems from the fact that during the last two decades the number of patients suffering from obesity has almost tripled. Unsurprisingly, also the number of bariatric surgeries, i.e., the ultimate effective strategy in the fight with morbid obesity, has increased. Amongst the important contributors to the obesity epidemic are modifiable exogenous factors such as poor nutritional habits, the consumption of high-caloric diets, improperly composed meals and low level of physical activity. The higher mortality rate observed in morbid obesity is attributable to the effects of CVD, a condition occurring in obese individuals much more often than in the general population [2].

Methods ordinarily used for permanent weight reduction, i.e., lifestyle modifications and/or pharmacotherapy, are usually ineffective in the case of individuals with morbid obesity (body mass index (BMI) >40 kg/m^2^). Nowadays, many surgical therapies are put forward for patients with BMI >40 kg/m^2^ or for individuals with BMI >35 kg/m^2^ and accompanying comorbidities (usually including arterial hypertension, type 2 diabetes mellitus or obstructive sleep apnea). Furthermore, surgical procedures are currently the most effective management technique, allowing for an extensive and permanent reduction in body weight. The above, in turn, is frequently accompanied by abatement in obesity-related comorbidities [3]. Finally, bariatric surgery stands out from other non-invasive methods with respect to the observed reduction in the number of CVD-related deaths in obese individuals. It has been demonstrated that lifestyle modifications inhibit the progression of diabetes and its complications [4]; they do not, however, reduce the rate of CVD events (this seems to be true even in the case of 10 to 20 year follow-ups) [5,6]. Similarly, lifestyle intervention together with medications neither improved primary CVD end points [7] nor did they decrease the incidence of CVD occurrence [8]. Therefore, in the case of individuals affected by morbid obesity and its complications, resorting to bariatric surgical procedures should not be considered as a renunciation of properly established medical treatment.

Bariatric surgery is a collective term encompassing many medical procedures. The most common of them are: sleeve gastrectomy (SG), Roux-en-Y gastric bypass (RYGB) and biliopancreatic diversion together with duodenal switch (BPD-DS). The aforementioned techniques range from completely restrictive (i.e., reducing the size of the stomach) to largely malabsorptive (i.e., decreasing the absorption of nutrients) methods. Moreover, it seems that the mechanisms of systemic metabolic improvement may differ depending on the applied method of bariatric surgery. SG is currently the most commonly performed bariatric technique involving the removal of ca. 80% of the stomach (mainly the fundus and the body alongside the arc of greater curvature) [9,10]. As a weight loss procedure, SG was first performed laparoscopically in 1999 as a part of the BPD-duodenal switch procedure [11]. Nowadays, laparoscopic SG (LSG) is a routinely applied procedure for high-risk, extremely obese individuals (due to its relatively low level of invasiveness and high effectiveness) [12,13]. The popularity of LSG is easily discernible. A study of Michigan Bariatric Surgery (MBSC) demonstrated that BPD-DS constituted only a small fraction (<1%) of all bariatric procedures performed in 2013 in the state of Michigan; LSG, on the other hand, accounted for over 2/3 (67.3%) of the total [14].

Bariatric surgical procedures exert local and systemic metabolic effects. The study performed by Heffron et al. demonstrated significant improvements in total cholesterol (TC), low-density lipoprotein cholesterol (LDL-C), triacylglycerols (TAG) and high-density lipoprotein cholesterol (HDL-C) levels after the application of bariatric surgery techniques in comparison to non-operated controls. However, the discussed alterations differed significantly depending on the type of the surgical procedure [15]. The aforementioned findings emphasize the need for further research in the area of metabolic effects exerted by bariatric procedures. This seems to be true especially for SG, a technique commonly used, though, paradoxically, it is the least extensively studied. 

It has been established that bariatric surgery lowers blood plasma lipids levels, mainly from TC, LDL-C and TAG fractions [15]. However, the knowledge from the field is far from complete. To date, the effect of sleeve gastrectomy on the contents of FFA, DAG and TAG in the liver and blood plasma has not been extensively studied. Moreover, also the influence of SG on the liver’s and plasma’s PCSK 9 level is unknown. The latter, though, seems to be worth studying, since the literature points to the relationship between a high intrahepatic proprotein convertase subtilisin/kexin type 9 (PCSK9) concentration and greater FFA and TAG storage in the tissue. This in turn increases the secretion of these lipids into the blood and therefore may exert systemic effects [16]. Given the above, we decided to exploit this research venue in the current investigation. Our initial assumption was that both high-fat diet (HFD) and SG may influence the liver PCSK9 level. This in turn, should be associated with the tissue lipid contents (FFA, TAG, DAG), and thus their blood plasma levels.

The data presented in the studies of Teslovich et al. and Abifadel et al. point to a crucial role of proprotein convertase subtilisin/kexin type 9 (PCSK9) in the regulation of the blood plasma cholesterol level [17,18]. PSCK9 is a compound protein expressed predominantly in the liver, small intestine, kidneys and brain. It belongs to a family of proprotein convertases. It causes lysosomal degradation of the low-density lipoprotein receptor (LDLR), resulting in its decreased density on the cell surface of hepatocytes [19]. Since the receptor mediates endocytosis in cholesterol-rich LDL, the above leads to an increased concentration of LDL-C in the circulation. However, the mechanism by which PCSK9 triggers the degradation of LDLR in hepatocytes remains unexplored. A possible explanation might be the ability of PCSK9 to selectively suppresses LDLR in the liver. This in turn prevents the immediate re-uptake of nascent VLDL (very low density lipoproteins) particles, and thus advances their shipment to peripheral tissues [20]. Moreover, it has been revealed that an increased plasma concentration of PCSK9 is associated with a somewhat greater number of CVD cases in elderly patients. Interestingly, this association appears to be true even after adjusting the model for other well-known risk factors for CVD [21]. 

So far, the regulation of the PCSK9 level in the liver has not been thoroughly studied. Nevertheless, the study of Miao et al. demonstrated that insulin increased PCSK9 mRNA and protein expression in hepatoma cells and primary rat hepatocytes [22]. These data are consistent with those from other previously published studies [23,24]. Moreover, insulin may also increase the mRNA level of LDL receptor in hepatoma cells of rats [25]. 

Despite the fact that studies published so far indicate that insulin plays a key regulatory role in the modulation of PCSK9 expression, it has been postulated that other factors may be of importance as well [22]. It was demonstrated, however, that one year of lifestyle changes (based on the increase of one’s physical activity and the introduction of healthy nutritional habits) failed to affect plasma PCSK9 concentration in a group of individuals with visceral obesity [26]. Moreover, the results obtained by Boyer et al. indicated that bariatric surgery (BPD-DS) contributes to reductions in the levels of circulating LDL-C and PCSK9 [27]. 

In summary, the literature analysis indicated that there are no reports on the impact of SG on PCSK9 levels in connection with the concentrations of free fatty acids (FFA), triacylglycerols (TAG) and diacylglycerols (DAG) in blood plasma. Moreover, data concerning the effect of bariatric surgery on intrahepatic lipids concentrations and PCSK9 content also seem to be missing. Therefore, our aim was to exploit this research venue in the current investigation. We hypothesized that both HFD and SG may influence liver PCSK9 levels. This in turn should be associated with the tissue lipid contents (FFA, TAG, DAG), and thus their blood plasma levels. 

## 2. Materials and Methods 

### 2.1. Animals

All research procedures were conducted in line with the recommendations contained in the “European Convention for the Protection of Vertebrate Animals Used for Experimental and Other Scientific Purposes”. Moreover, they were approved by the Medical University of Bialystok’s Ethical Committee for Animal Experiments (protocol number 89/2017, 9 June 2017).

For the purposes of this investigation, thirty-four male Wistar rats (initial age: ~4 weeks, initial body weight: 70–90 g) were obtained from a licensed breeder. The animals were maintained in the appropriate conditions (i.e., temperature: 21–22 °C, circadian rhythm: 12 h/12 h reversed light/dark cycle, with light on ≥06:00 a.m.). During the course of the study, the animals had unlimited (ad libitum) access to water and fodder. At the beginning of the study, random allocation of the rodents into one of the following groups was performed:

* Control, *n* = 10;
* HFD, *n* = 12;
* Bariatric surgery (BS), *n* = 12.

The animals from the control group (Control) received a standard rodent fodder (Table 1) over a time span of 10 weeks (Table 1). HFD and BS rats, on the other hand, ate fat-rich fodder (Table 2) for the equivalent period of time (10 weeks) (Table 2). 

Food consumption as well as the animals’ body mass were recorded every third day (Table 3).

After 10 weeks and an overnight fast, the animals from the control and high-fat diet groups were anesthetized by sodium phenobarbital (80 mg per kg of body weight) injection into the peritoneum. Next, samples of blood from the tail were drawn in order to determine the fasting blood glucose level (using an Accu-Chek glucometer, Roche, Bayer, Germany). Moreover, abdominal aorta blood samples were also gathered. To obtain blood plasma, the body fluid samples were placed into glass tubes. In order to prevent blood coagulation, sodium heparin was added to the tubes. Next, the samples underwent 10 min centrifugation with a speed of 3000 *g* (temperature: 4 °C). The resultant samples were then precooled and stocked at −80 °C until future assessments. 

Liver tissue was rinsed with cold phosphate-buffered saline (PBS; Cm = 0.02 M, pH: 7.4) and dried. 

Then, liver samples were extracted, freeze-clamped with aluminum tongs (precooled in liquid nitrogen) and stored at a temperature of −80 °C (until further analyses) or fixed with formalin, embedded in paraffin and stained with hematoxylin-eosin (HE).

The rats from the BS group (*n* = 12) underwent SG surgery. Following the procedure, all rats were treated with intravenous hydration and given clear water for the first 3 days. After that period, a slow adjustment (over another 4 weeks) to a standard rodent fodder (Agropol, Motycz, Poland) was conducted.

Then, after 4 weeks of the experiment, the animals from the SG group were sacrificed. Liver tissue and blood plasma were collected in the same way as described for the HFD and control groups. The samples were stored at −80 °C until further analyses.

### 2.2. Preparation of Homogenates

On the day of the analysis, liver tissue was thawed on ice, weighed and cleansed with PBS (pH = 7.4). Afterward, homogenization took place. It was performed using a glass homogenizer (Omni International, Kennesaw, GA, USA) whilst all the samples were kept on ice. Subsequently, the resulting suspension was sonicated using an ultrasonic homogenizer (cell disrupter) (1800 J per sample, 20 s three times on ice) (UP 400S, Hielscher, Teltow, Germany). Next, the homogenated tissue underwent centrifugation for 30 min at a temperature of 4 °C and a speed of 12,000 *g*. The resultant supernatant was further analyzed.

### 2.3. Assessment of Total Protein Concentration

The concentration of PCSK9 in the liver and plasma was determined by applying commercial ELISA kits in line with the attached manufacturer’s instructions. The kits were provided by Elabscience Biotechnology Inc. (catalog number: E-EL-R2487; Houston, TX, USA) and CUSABIO (catalog number: CSB-EL017647RA; Wuhan, China) for the liver and plasma samples, respectively. The detection limits of the kits were 0.78–50 ng/mL and 93.75–6000 ng/mL (for the liver and blood plasma, respectively) with intra-assay coefficient variation <8% and inter-assay coefficient variation <10%. The absorbance was measured in a microplate reader (ELx800, Bio Tek Instruments, Winooski, VT, USA) at 450 nm and all assays were performed in duplicate.

### 2.4. Surgical Procedure

All rats were fasted overnight before surgery. On the day of the procedure (sleeve gastrectomy), the rodents were anesthetized and received an antibiotic—ciprofloxacin (0.1 mg per kg of body weight via injection into the peritoneum)—10 min prior to the initiation of the operation. At the beginning of the surgical procedure, a 2 cm incision in the midline was performed. The stomach was identified and carefully displaced from the abdominopelvic cavity. In further steps of the procedure, the lateral part of the stomach (~80% of the total stomach volume) was resected. The above led to the creation of a tubular gastric piece connecting the esophagus with the duodenum. The newly formed gastric sleeve was softly inserted, and the abdominopelvical cavity was subsequently sealed using a continuous stitch of silk sutures. After the surgery, all the rodents received suitable postoperative care, including antibiotics, painkillers and subcutaneous hydration for the next 72 h. Solid diet was introduced 3 days after the surgery. The rats were then permitted to recuperate until their body mass was stable. 

### 2.5. Plasma Lipid Concentrations

Lipid compositions (FFA, DAG, TAG) in the blood plasma and liver were measured in line with a method specified in a paper by Glaser and co-workers [30]. Briefly, lipid species of interest (FFA, DAG, TAG) were extracted from blood plasma based on the Folch protocol [31]. Next, methanolic HCl was administered to the samples. The mixture was then warmed for 45 min to the temperature of 85 °C so that methyl esters could be synthesized. Upon cooling, 1 mL hexane was added. Then the tubes underwent 5 min centrifugation at the speed of 900 *g*. The supernatant was moved to fresh glass test tubes. The samples were then evaporated using nitrogen flow. The remaining lipid extracts were resuspended in hexane (50 µL). Finally, GLC (Hewlett-Packard 5890 Series II gas chromatograph, HP-INNOWax capillary column) was applied to identify and measure the volume of the obtained fatty acid methyl esters. Identification was made possible by comparing the measurements with the retention times of commercially available standards.

### 2.6. Statistical Analyses

The obtained data were analyzed using GraphPad Prism 5. First, the data were assessed with respect to the normality of distributions and homogeneity of variances. If they positively passed both tests they underwent one-way ANOVA. As a post-hoc analysis, Tukey’s Honestly Significance Difference (HSD) test was applied. Alternatively, if the assumptions of the abovementioned procedures did not hold, one-way ANOVA of ranks (Kruskal–Wallis test) with the following Wilcoxon’s pairwise tests were performed. Statistical differences were acknowledged only when the obtained *p*-values were below 0.05. The data are presented as the average ± standard deviation (SD).

## 3. Results

### 3.1. Plasma and Liver PCSK9 Concentrations.

**Figure 1:**
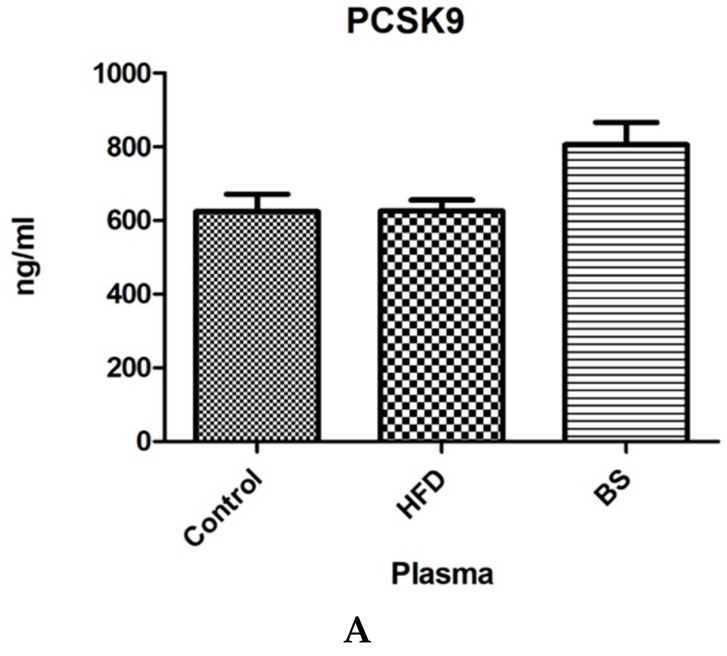

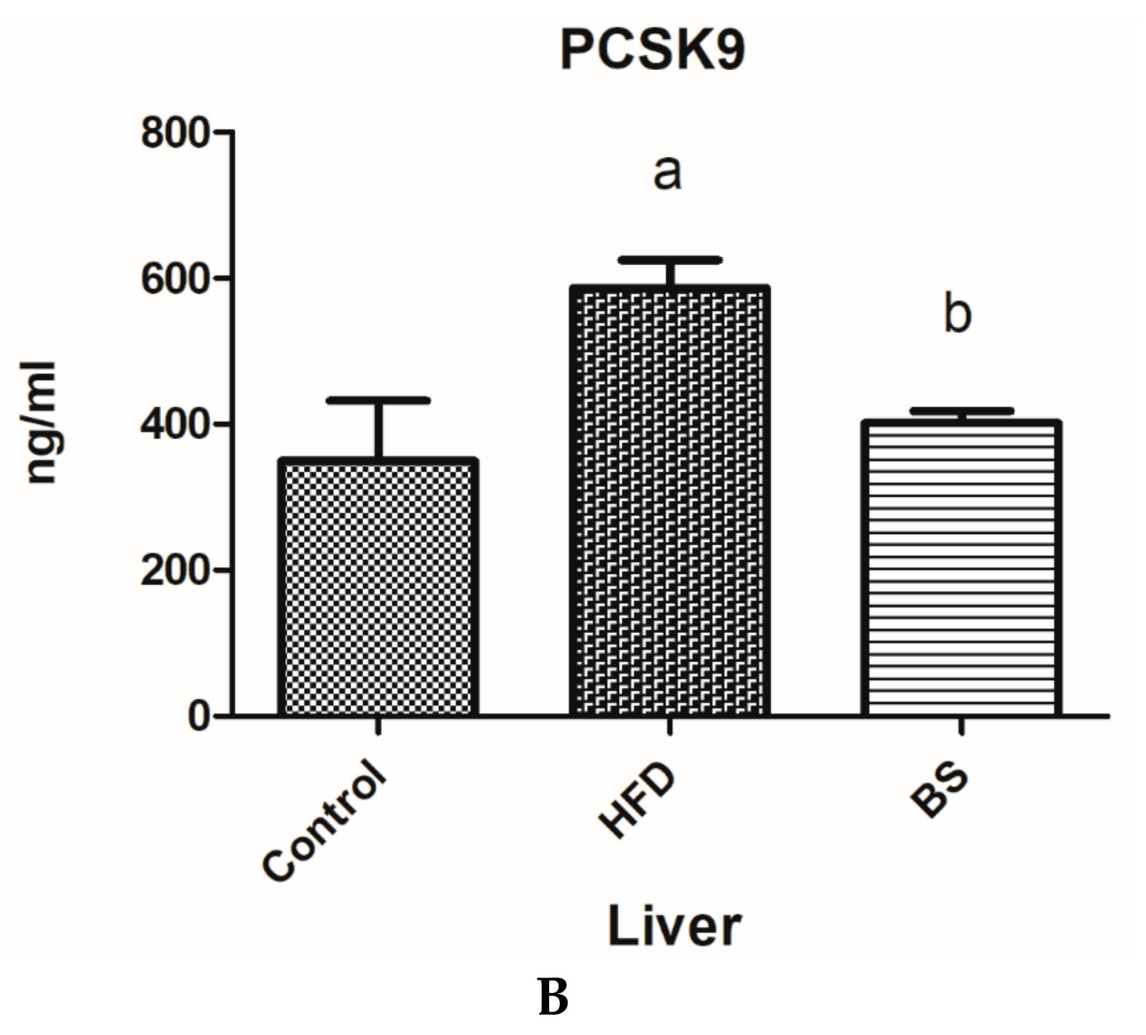
Concentrations of PCSK9 (ng/mL) in blood plasma (**A**) and liver tissue (**B**). Bar heights correspond to averages and error bars represent standard deviations. Statistical significance markers: a—vs. Control (*p* < 0.05), b—vs. HFD (*p* < 0.05).

In blood plasma, the concentration of PCSK9 showed an upward trend in the rats after bariatric surgery (*p* > 0.05; Figure 1A) in comparison to the control animals. We did not observe any difference in the plasma concentration of PCSK9 in the HFD group compared to the control group. In the liver, the concentration of PCSK9 was significantly higher in the rats receiving HFD (*p* < 0.05; Figure 1B) as compared to the control group. Moreover, the liver’s concentration of PCSK9 was significantly lower in the group after bariatric surgery in comparison with the HFD group (*p* < 0.05).

**Figure 2:**
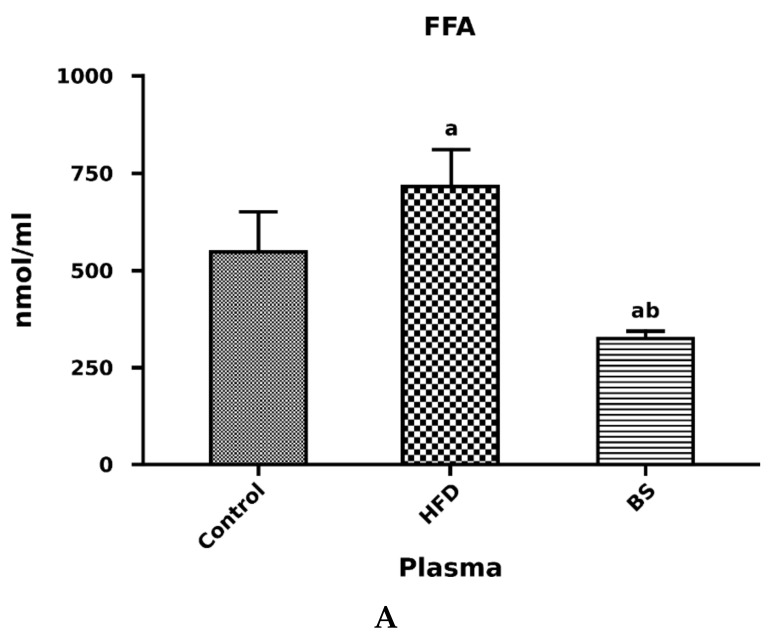

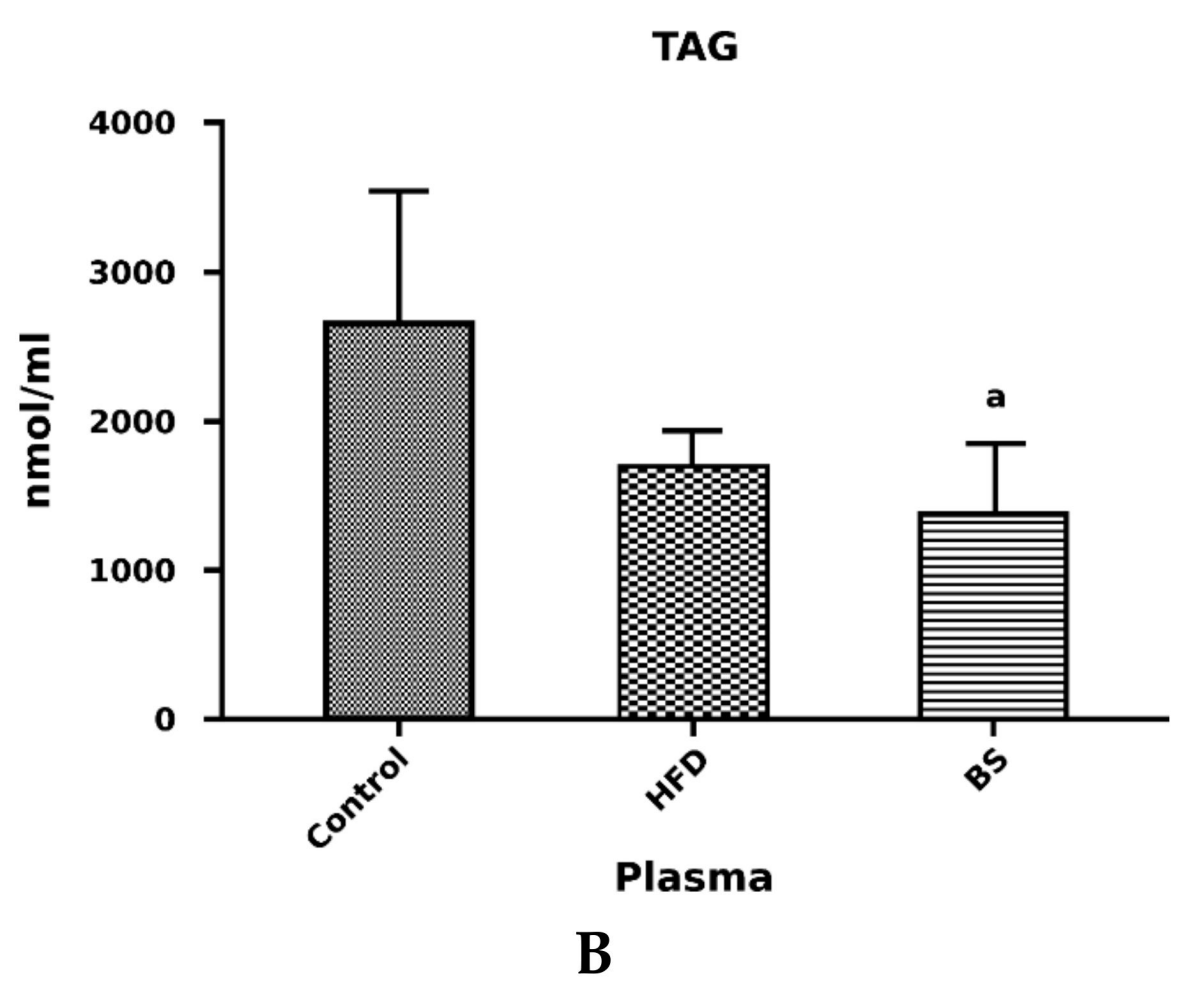
Concentrations of free fatty acids (FFA) (nmol/mL) (**A**) and triacylglycerols (TAG) (nmol/mL) (**B**) in blood plasma. Bar heights correspond to averages and error bars represent standard deviations. Statistical significance markers: a—vs. Control (*p* < 0.05), b—vs. HFD (*p* < 0.05).

**Figure 3:**
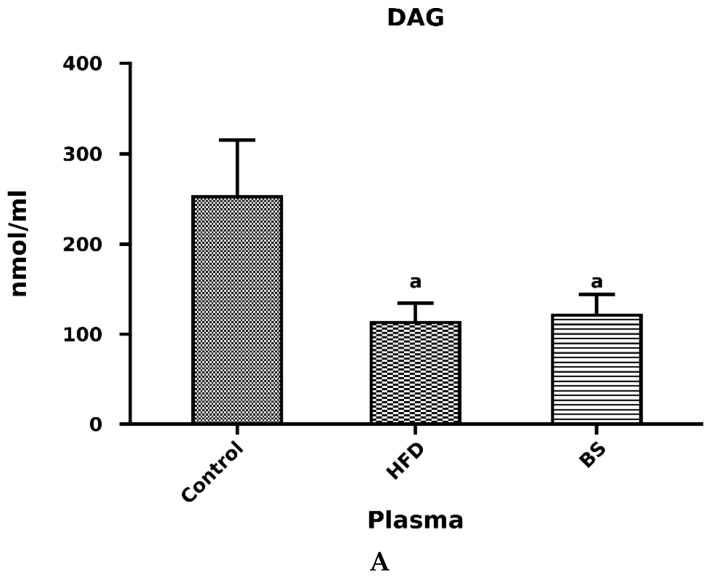

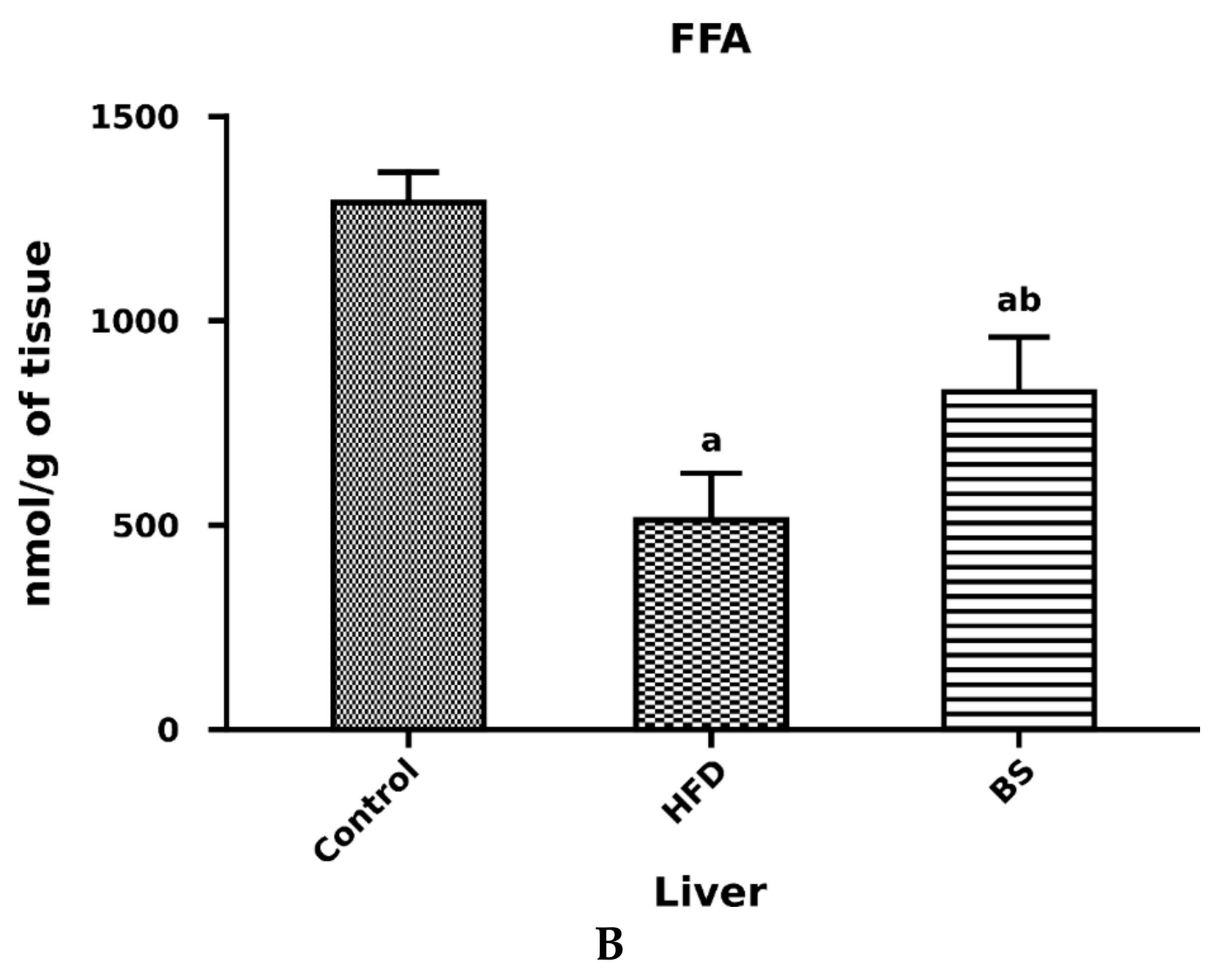
Concentrations of diacylglycerols (DAG) (nmol/mL) in blood plasma (**A**) and FFA (nmol/g of tissue) in the liver (**B**). Bar heights correspond to averages and error bars represent standard deviations. Statistical significance markers: a—vs. Control (*p* < 0.05), b—vs. HFD (*p* < 0.05).

We noticed a significant decrease in the blood plasma concentration of FFA in the rats after bariatric surgery as compared to the control group. Additionally, there was a significant drop in the blood plasma concentration of FFA in the BS group compared to the HFD group (*p* < 0.05, Figure 2A). The free fatty acid concentration in the plasma was markedly elevated in the HFD group in comparison with the control group (*p* < 0.05, Figure 2A). We did not observe any difference in TAG level in the high-fat diet group as compared to the control one (*p* > 0.05, Figure 2B), but we noticed a significant decrease in TAG contents in the post bariatric surgery group in comparison with the control group (*p* < 0.05, Figure 2B). We also noticed a considerable decrease in the blood plasma total DAG concentration in the HFD rats in relation to the control rats (*p* <0.05, Figure 3A). Additionally, the blood plasma concentration of DAG was significantly decreased in the BS group compared to the control group.

### 3.2. Liver Total FFA, TAG and DAG Concentrations 

**Figure 4:**
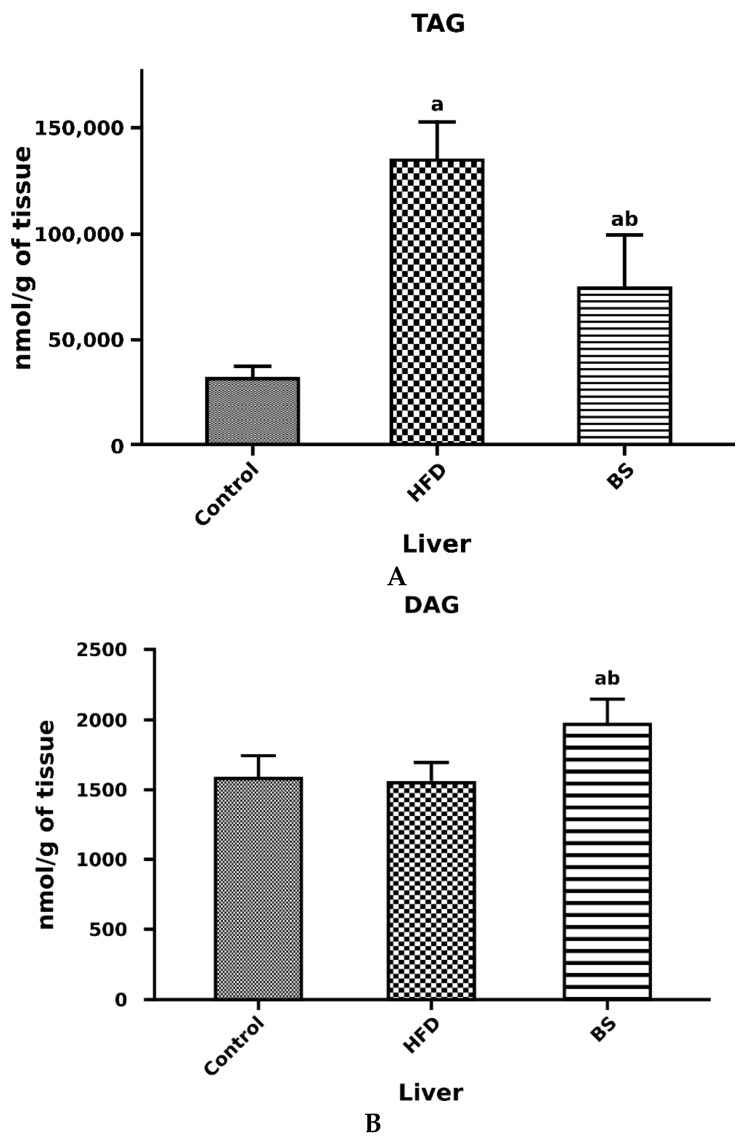
Concentrations of TAG (nmol/g of tissue) (**A**) and DAG (nmol/g of tissue) (**B**) in the liver. Bar heights correspond to averages and error bars represent standard deviations. Statistical significance markers: a—vs. Control (*p* < 0.05), b—vs. HFD (*p* < 0.05).

We observed a significant decrease in the liver concentration of FFA in rats after bariatric surgery as compared to the control group. On the other hand, the total concentration of FFA in the liver of rats after bariatric surgery was markedly elevated when compared to the HFD group. Interestingly, the total FFA concentration was significantly decreased in the HFD group as compared to the control group (*p* < 0.05, Figure 3B). The total TAG concentration in the liver was markedly elevated in the HFD group in comparison with the control group (*p* < 0.05, Figure 4A). Additionally, the total concentration of TAG in the liver was increased in the BS group in relation to the control group (*p* < 0.05, Figure 4B). We also noticed a considerable decrease in the total hepatic TAG concentration in the BS group in comparison to the HFD group (*p* < 0.05, Figure 4A). The hepatic concentration of DAG was significantly increased in the BS group in comparison with the control and HFD groups (*p* < 0.05, Figure 4B).

### 3.3. Histopathological Evaluation

**Figure 5:**
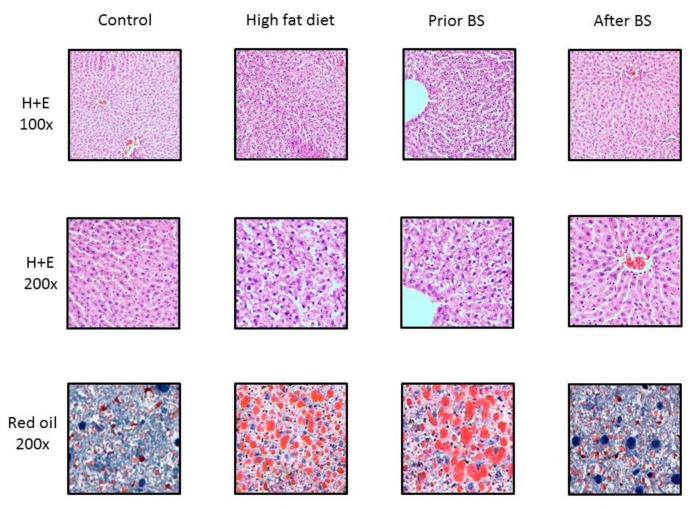
Effect of high-fat-diet feeding and bariatric surgery on hepatic steatosis. The photos were made at 100× and 200× magnifications. Staining was performed using hematoxilin and eosin (H + E) and red oil. BS—bariatric surgery.

Tissue samples obtained from the livers of the rodents fed a high-fat diet (HFD) presented symptoms of steatosis upon histological evaluation (Figure 5) after hematoxylin and eosin staining. Figure 5 clearly depicts lipid droplets accumulating in the hepatocytes. Moreover, mild derangement of the hepatic architecture also seemed to be noticeable in the HFD rats. Interestingly, bariatric treatment seemed to reverse the abovementioned steatosis, since we noticed virtually no differences in histopathological specimens between the samples coming from rats in the control and BS groups.

## 4. Discussion

Obesity and its related health complications, such as insulin resistance, type 2 diabetes, hypertension, cardiovascular disease (CVD) and some types of cancer, have become significant contributors to the overall mortality in developed countries. The main reason for the increasing amount of obese people is high-fat diet and sedentary lifestyle in conjunction with simple access to energy-rich food in genetically predisposed individuals [32]. Obesity and HFD contribute to significantly increased concentrations of FFA in the blood plasma. This, in turn, leads to excessive FFA uptake by some metabolically active tissues, such as the liver, adipose tissue and/or skeletal muscle. The increased FFA influx exceeds the oxidative capacity of the cells and, therefore, leads to an excessive accumulation of intracellular lipids. This increase in lipid content negatively affects the metabolic function of tissues. Moreover, the manner by which lipids affect cellular metabolism seems to be tissue-specific. Excessive accumulation of FFA, DAG and/or TAG, for instance, inhibits the insulin signal transduction pathway in skeletal muscles and boosts the rate of gluconeogenesis in the liver. The latter is a key regulator of the body’s glucose and lipid metabolism. Obesity and high-fat diet (HFD) are connected to excessive intracellular lipid accretion. It appears that even a relatively short-term consumption of HFD may result in hepatic fat accumulation and the organ’s insulin resistance (IR) in rodents [33]. Insulin resistance favors adipocyte-origin lipolysis. This in turn contributes to increased hepatic free fatty acid (FFA) uptake. Moreover, insulin resistance-associated hyperinsulinemia stimulates hepatic de novo (e.g., from carbohydrates) lipogenesis, thus further increasing their accumulation in the liver.

The data obtained by Miao et al. [22] indicated that insulin may directly stimulate PCSK9 expression. This was confirmed by observations in different models of diabetes, e.g., liver insulin receptor knockout (LIRKO) rodents, *ob*/*ob* mice treated with insulin receptor—IR antisense oligonucleotides—ASO (IR ASO) or mice after streptozotocin (STZ) injection, all of which were characterized by decreased levels of Pcsk9. Nevertheless, the very same data suggest that insulin is not necessarily a major governing agent for PCSK9 expression. The aforementioned LIRKO mice, for instance, despite their evident lack of insulin responsiveness, showed ~80% drop in PCSK9 upon fasting. Furthermore, Costet et al. [23] speculated that PCSK9 plays an important role in dyslipidemias occurring in insulin-related pathophysiological states such as insulin resistance and/or diabetes. 

The purpose of the current investigation was to obtain more insight into the changes of PCSK9 levels and lipid concentrations in the liver and blood plasma of rats after bariatric surgery. Our findings suggest that SG can positively influence the concentration of plasma PCSK9 in rats. We noticed a trend towards an increase in the PCSK9 level in rats after SG. This is consistent with the results of Boyer et al. [27], although they presented a significant increase in PCSK 9 levels in the blood plasma of patients after BPD-DS in the acute phase. However, the biological mechanisms crucial to this observation are not known. We speculate that SG could induce an inflammatory response. Boyer et al. [27] also noticed that in the chronic phase of post-bariatric surgery (BPD-DS), the level of PCSK9 in blood plasma was significantly decreased. This finding could be attributed to the long-term weight loss stemming from limited calorie consumption after BPD-DS surgery. In the current investigation, we found that SG surgery affected the level of PCSK9 in the liver of rats. We observed a significant decrease in the liver PCSK9 levels in the post-bariatric surgery group in comparison with the HFD group. This made the SG rats similar to the control group, at least with respect to the PCSK9 protein level. Interestingly, a study by Dettlaff-Pokora et al. [34] indicated that a partial surgical removal of adipose tissue (i.e., white adipose tissue (WAT)) in rats may cause an upregulation of liver PCSK9-encoding genes. This probably contributed to an elevated level of circulating PCSK9 and a decrease in the liver LDLR expression. Furthermore, the aforementioned procedure [35] was associated with the concomitant upregulation of genes encoding proteins engaged in TAG synthesis/assembly and secretion, and therefore it also upregulated the concentrations of such proteins. In our study, we showed a significant decrease in the liver concentration of TAG in the case of rats that had undergone bariatric surgery. This was accompanied by a decrease in TAG concentration in their blood plasma. The available literature data indicate the existence of a positive correlation between a high intrahepatic PCSK9 concentration and increased adipose and hepatic tissue lipid storage (FFA, TAG), as well as the secretion of TAG into the blood plasma [16]. We speculate that a significant decrease in the liver concentration of PCSK9, as observed in our study, could cause a decrease in the total TAG concentration in the liver and blood plasma. In the meta-analysis of 178 studies of bariatric surgical techniques, Heffron et al. [15] noticed a significant reduction in the average TAG level in surgical subjects compared with the baseline condition (−61.6 mg/dL), although this varied highly depending on the procedure. Interestingly, Ruscica et al. [36] observed a strong positive correlation between liver fat accumulation and the level of circulating PCSK9. The above was apparent in patients suspected for liver disease (NASH) and in those submitted to bariatric surgery. Significantly, the association between steatosis and PCSK9 level was uninfluenced by necroinflammation and hepatocellular ballooning. This last piece of information points out that PCKS9 is induced independent of inflammatory cytokine action, but in direct connection to the liver TAG content [36].

In our study, we only found a statistically insignificant increase in the PCSK 9 plasma concentration after bariatric surgery, while in a study by Ghanim et al. [37] a similar but statistically significant increase was observed. This is rather surprising, although still possible to reconcile. The difference observed by us was in the range of +30% (Figure 1) and did not reach statistical significance due to the high intra-group variation. We postulate that such a difference could reach the significance level if a larger number of animals was employed in the experiment. In addition, the two studies in question (Ghanim and ours) differ with respect to the applied procedure (SG vs. RYGB), which could exert slightly different effects on PCSK9 plasma levels. Still, based on the discussed results, it is difficult to state for sure whether sleeve gastrectomy has a direct effect on the release of PCSK9 from the liver. Only further scientific investigation can bring us closer to the truth.

Interestingly, the association between the serum concentrations of TAG and PCSK9 was indicated in healthy children and adolescents [25,38,39], as well as in healthy adults [39,40]. Additionally, circulating PCSK9 and VLDL-triacylglycerol concentrations in patients with diabetes have been demonstrated to decrease concomitantly in response to fenofibrate treatment [41]. Furthermore, the results of experiments on PCSK9 null mice suggest that PCSK9 deficiency may contribute to decreased postprandial triglyceridemia [42]. Moreover, Lambert et al. noticed that an overexpression of PCSK9 in mice was associated with an increase in VLDL synthesis [43]. All of the abovementioned data point to the possible association between circulating TAG and PCSK9 concentrations in both physiological and pathological conditions. However, the specific molecular mechanism behind this association remains unexplained.

In our work, we found that the plasma concentration of FFA significantly decreased in the SG group compared to the control and HFD groups. These data are consistent with the results of a study by Kawano et al. [44], who showed that the plasma concentration of FFA in rats was significantly lower in the group that underwent sleeve gastrectomy than in both the gastric banding and sham groups. They also found that the TAG concentration in the liver of SG rats was significantly lower compared with those in the other two groups. We also observed a decreased concentration of TAG in the liver of rats after bariatric surgery, as compared to the control and HFD groups. These data imply that SG can ameliorate lipid metabolism in the liver and blood plasma. We further demonstrated that bariatric surgery significantly reduced the DAG concentration in blood plasma. Additionally, we noticed that the liver concentration of DAG was significantly decreased in the HFD group as compared with the bariatric surgery group. The above is inconsistent with a study of Zabielski et al. [45] that showed that HFD leads to increased DAG accumulation. Further studies are needed to explain the impact of bariatric surgery on the content of DAG in the liver. There is only a limited amount of data regarding the impact of bariatric surgery on the concentration of bioactive lipids (such as FFA, TAG and DAG) in the liver and blood plasma. 

Taken together, our study provides additional information on PCSK9 levels after bariatric surgery. Moreover, it underscores the possibility that sleeve gastrectomy reduces the level of PCSK9 in the liver and circulation, in combination with decreased concentrations of FFA and TAG. 

## Figures and Tables

**Table 1:** Nutritional composition of the standard rodent fodder used throughout the study—Provider: Agropol ([28], LSM diet).

Ingredient	Control
Protein, min (%)	23
Fat, min (%)	3
Ash, max (%)	7.5
Fiber, max (%)	5
Lysine, max (%)	1.5
Methionine + cysteine, min (%)	0.8
Calcium, min (%)	1.1
Phosphorus, min (%)	0.7
Sodium, max (%)	0.2
Vitamin A (mg/kg)	8000
Vitamin B3 (mg/kg)	1000
Vitamin E (mg/kg)	50

**Table 2:** Nutritional composition of the fat-rich animal fodder (high-fat diet) used throughout the study—Provider: Research Diets Inc. ([29], #D12492).

Nutrient Class	Ingredient	High-Fat Diet (gm %)
Protein	Casein	25.8
	Cystein L	0.4
Carbohydrate	Lodex 10	16.2
	Sucrose	9.4
	Corn Starch	0
	Maltodextrin	0
Fiber	Solka Floc, FCC200	6.5
	Celulose	0
Fat	Lard	31.7
	Soybean oil, USP	3.2
Mineral	S10026	6.5
Vitamin	Choline bitartrate	0.3
	Vitamin mix	0.1

**Table 3:** Basal biological parameters of the rats. * *p* < 0.05 vs. Control.

Variable	Control	High-Fat Diet (HFD)	Bariatric Surgery (BS)
Initial body weight (g)	227.7 ± 14.8	227.7 ± 14.8	227.7 ± 14.8
Body weight prior to BS (g)	286.3 ± 14.7	392.8 ± 28.3 *	380.2 ± 32.6 *
Body weight after BS (g)	302.4 ± 16.8	405.6 ± 49.4 *	258.4 ± 19.3 *
Daily food intake prior to BS (g)	13.7 ± 3.5	16.3 ± 5.6	15.7 ± 4.3
Daily food intake after BS (g)	NA	NA	9.6 ± 3.9
